# Germination ecology of *Chloris truncata* and its implication for weed management

**DOI:** 10.1371/journal.pone.0199949

**Published:** 2018-07-09

**Authors:** Bhagirath Singh Chauhan, Sudheesh Manalil, Singarayer Florentine, Prashant Jha

**Affiliations:** 1 The Centre for Crop Science, Queensland Alliance for Agriculture and Food Innovation (QAAFI), The University of Queensland, Gatton, Queensland, Australia; 2 School of Agriculture and Environment, Institute of Agriculture, The University of Western Australia, Perth, Australia; 3 Amrita University, Coimbatore, India; 4 Centre for Environmental Management, Faculty of Science and Technology, Federation University Australia, Mount Helen, Victoria, Australia; 5 Southern Agricultural Research Centre, Montana State University, Huntley, Montana, United States of America; College of Agricultural Sciences, UNITED STATES

## Abstract

*Chloris truncata* is a significant weed in summer crops in the subtropical region of Australia. A study was conducted to evaluate the effect of environmental factors on germination and emergence of two populations of *C*. *truncata*. Overall, germination was not affected by the populations. Seeds germinated at a wide range of alternating day/night temperatures, suggesting that seeds can germinate throughout the spring, winter and autumn seasons. Seed germination was stimulated by the presence of light; however, 51 to 71% of these seeds still germinated in the dark. The sodium chloride concentration and osmotic potential required to inhibit germination of 50% of the population were 179 mM and -0.52 MPa, respectively. A high proportion of seeds germinated over a wide pH range (4 to 10). Seeds placed on the soil surface had greatest germination (67%) and a burial depth of 3 cm resulted in complete inhibition of emergence. The sorghum residue amount required to reduce emergence by 50% was 1.8 t ha^-1^. The results suggest that, although this weed will be favored in no-till systems, residue retention on the soil surface will help in reducing its infestation. Seed bank buildup can be managed by burying seeds below the depth of emergence.

## Introduction

Over the last few decades, weed management practices in Australia have changed from a regime of frequent cultivation to no-till systems with less soil-disturbance, and a reliance on glyphosate for pre-seeding and fallow weed control. These changed practices have resulted in a shift in weed species to those favored by no-till and reduced-till systems [1]. One such example is *Chloris truncata* R.Br., which has become a significant weed in summer grain crops and cotton in the subtropical region of Australia [2–4]. It is a C_4_ annual or short-lived perennial summer grass species, which is native to Australia and is found throughout the Australian mainland [2, 5].

In a survey of summer fallow fields in Western Australia, this species was found in 12% of the investigated sites [6], and *C*. *truncata* was found in 16% of the fields surveyed in cotton-growing regions of Queensland and New South Wales, covering 135 fields [7]. This species has been ranked in the top 10 most important summer fallow weeds in Australia, based on estimates of infested area, yield loss and revenue loss [3]. Also, most growers in Australia traditionally rely on residual moisture for planting their crops, and in such situations *C*. *truncata* may significantly reduce the yield of winter crops by utilizing residual soil moisture during summer [8]. A density of 36 plants/m^2^ of this weed has been found to reduce mungbean grain yield by 50% (Manalil and Chauhan, unpublished data).

As mentioned earlier, growers in Australia now predominantly rely on glyphosate for pre-sowing and fallow weed control. However, because of the over-reliance on this herbicide, several populations of *C*. *truncata* have evolved resistance to glyphosate [9]. This species has prolific seed production, and its seeds are dispersed via wind as the mature spike breaks from the plant [2]. These natural reproductive factors together with evolving herbicide resistance suggests that *C*. *truncata* will become an even more important and troublesome weed in Australia unless steps are taken to reduce its economic impact on crop production.

In order to reduce reliance on herbicide use and to carefully promote crop germination whilst hindering weed production, integrated weed management programs urgently need to be developed to manage weeds like *C*. *truncata*. However, the development of such programs will depend on a detailed understanding of seed germination biology for the competing species [10]. Seed germination is affected by several environmental factors, such as temperature, light, soil moisture, soil salinity, soil pH, crop residue (as mulch) and seed burial depth.

Whilst some information about the germination requirements of *C*. *truncata* is available for South Australian populations [11], little is available for Queensland populations. These two states of Australia have very different climatic conditions, which are known to affect seed germination biology of weeds [12]. In experiments conducted in South Australia, seed germination was strongly stimulated by light, with only 2% germination in the dark[11]. The germination requirements of *C*. *truncata* in Queensland appear to be somewhat different from those of *C*. *truncata* in South Australia. The seeds in the South Australian study were found to germinate over a wide temperature range (10 to 40°C), with maximum germination at 20 to 25°C [11]. However, constant temperature conditions rarely occur in nature, and studies using constant temperature conditions should be avoided in experiments [13]. We are aware that fluctuations in temperature can affect weed seed germination compared with seeds kept at constant temperature, but such important information is not available on *C*. *truncata*.

Similarly, the effect of burial depth and crop residue amount on seedling emergence of *C*. *truncata* is poorly understood in the northern regions of Australia. Seed burial depth can affect germination and emergence by moderating the availability of light, temperature, and moisture. Knowledge of the impact of burial depth on seedling emergence will increase confidence in the use of tillage systems to manage *C*. *truncata*. The use of crop residues such as mulch should also be a part of integrated weed management programs as this can provide weed suppression through its physical presence on the soil surface [14].

In addition, weed seeds which reach maturity in different environmental conditions may have different germination requirements, and in this respect a previous study reported different germination values for eight populations of *Spergula arvensis* L. [15]. With such potential uncertainty in mind, we believe that better understanding of seed germination and seedling emergence of *C*. *truncata* could contribute to the development of more suitable control measures based on our ability to predict this species’ potential for invasion into new areas. To contribute to this aim, the specific objectives of this study were to examine the effects of alternating temperature and light conditions, moisture stress, salinity, pH, burial depth and presence of sorghum residue on the germination and emergence of *C*. *truncata*.

## Results and discussion

### Temperature and light

**Fig 1:**
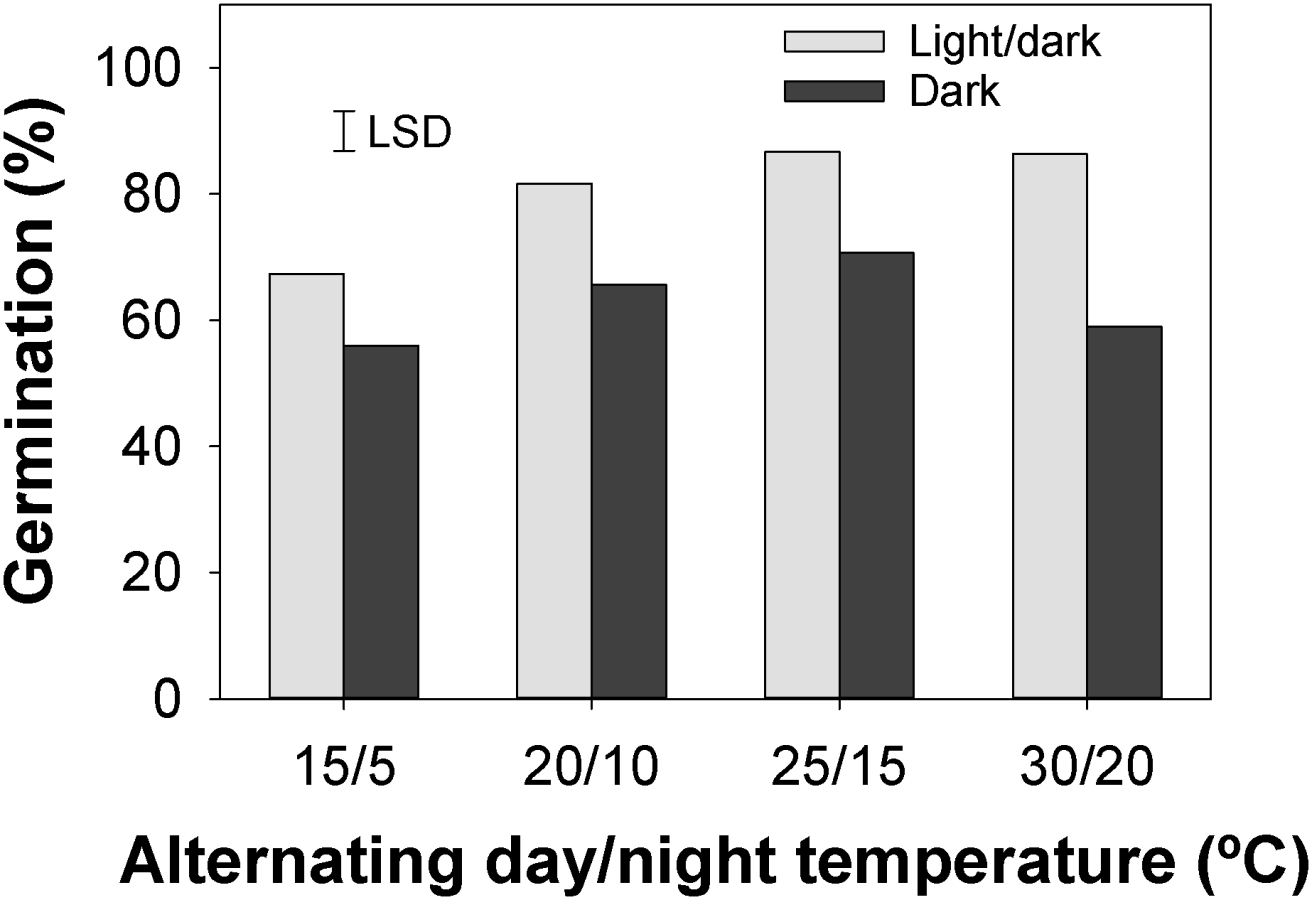
Effect of alternating day/night temperatures and light regimes on seed germination of *Chloris truncata* after 21 d of incubation.

The effect of the population on germination was non-significant, indicating that the prior history of the selected populations had no effect on our results. However, the interactive effect of temperature and light was highly significant for seed germination of *C*. *truncata*. Our results showed that whilst seed germination in light/dark conditions was highest (87%) at 25/15°C, it was statistically similar to the germination obtained at 20/10 and 30/20°C (Fig 1). In these light/dark conditions, the lowest germination rate (67%) was obtained at 15/5°C. In the dark regime, higher germination (66–71%) was obtained at 20/10 and 25/15°C than at 15/5°C (56%) and 30/20°C (59%).

Although there were some differences, seeds of *C*. *truncata* germinated at all the tested temperature regimes, indicating this species ability to germinate and emerge under field conditions throughout spring, summer, and autumn in Queensland. These results are consistent with the previous study conducted in South Australia, in which the authors suggested that *C*. *truncata* is likely to germinate after rainfall events in spring, summer, and autumn [11]. The authors also suggested that its ability to germinate in spring would make weed control difficult as winter cereal crops are still growing in the fields at that time. In our study, the optimum temperatures for maximum germination ranged from 20/10 to 30/20°C (average of 15 to 25°C). This concurred with a previous study in which the optimum temperatures were found to be from 15 to 30°C [11].

Whilst we found that seed germination was stimulated by the presence of light at all four temperature regimes, it was determined that 51 to 71% seeds still germinated in the dark. These results are very different to those reported for the South Australian populations, where germination ranged from 0 to 2% in the dark [11]. We suspect that these differences between the two studies could be due to different climatic environments (Queensland vs South Australia) experienced by the seeds, or possibly caused by seed age. Generally, a requirement for light implies that germination would occur only on or near the soil surface [10]. Our results also suggest that buried seeds are still capable of germination. It is suggested, nevertheless, that greater germination of *C*. *truncata* would occur on the surface than seeds buried in soil.

### Salt stress

**Fig 2:**
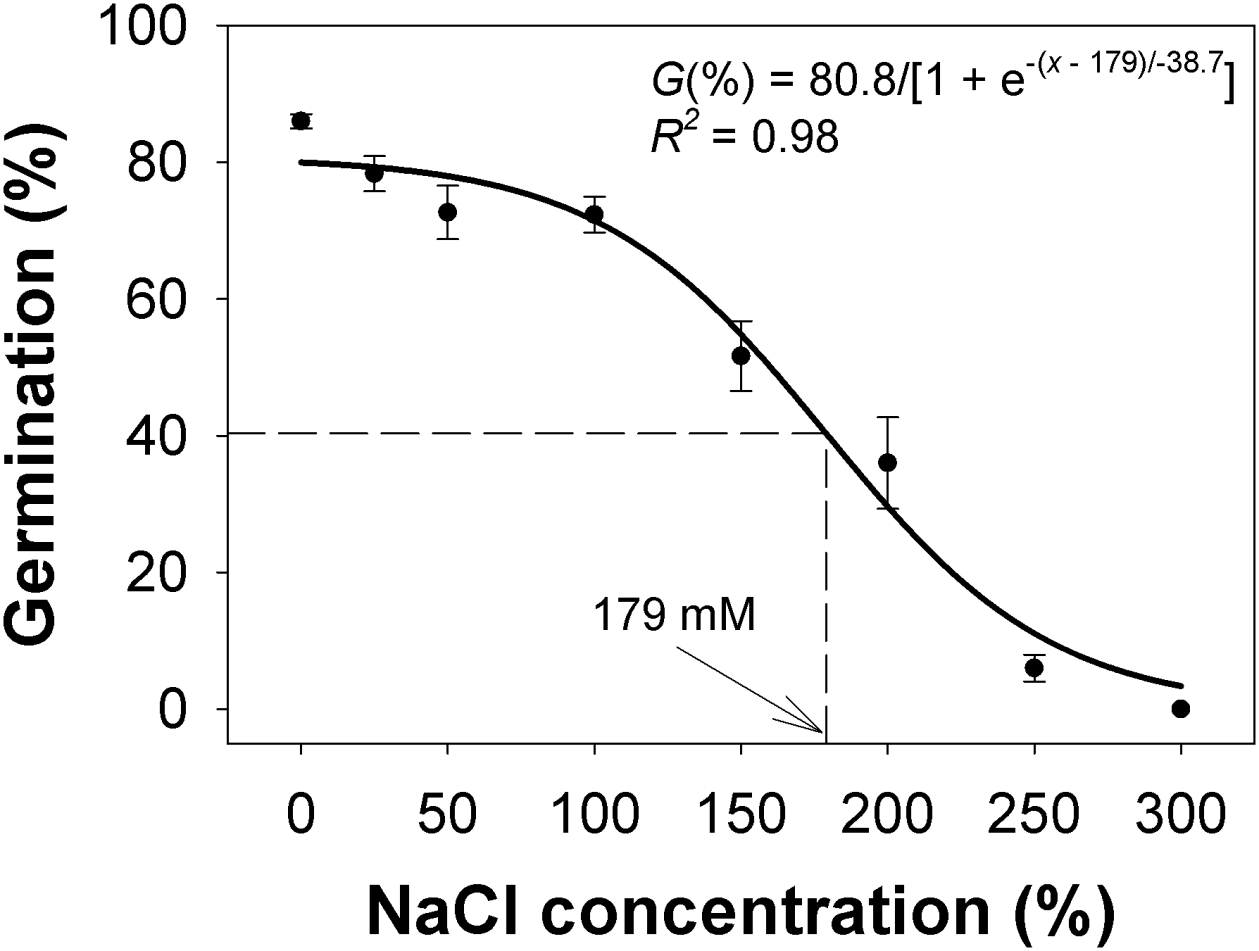
Effect of sodium chloride (NaCl) concentration on seed germination of *Chloris truncata* after 21 d of incubation at 25/15°C day/night temperature. The line represents a three-parameter sigmoid model fitted to the data. The arrow shows the NaCl concentration required to inhibit 50% germination. Vertical bars represent standard error of mean.

A sigmoid response was observed in the germination of *C*. *truncata* seeds at various salt concentrations (Fig 2). The results indicated that maximum germination (81%) was obtained in the control (no salt stress), but germination was still greater than 70% up to a concentration of 100 mM NaCl. Some seeds were observed to germinate at 250 mM NaCl, but seed germination was completely inhibited at 300 mM NaCl. In the previous study in South Australia, 250 mM NaCl completely inhibited germination of *C*. *truncata* [11]. In our latest study, the concentration of NaCl required to inhibit germination by 50% was 179 mM. This concentration was many times stronger than reported for the South Australian populations (51 to 73 mM NaCl), and these results strongly suggest that the Queensland populations may be significantly more tolerant to salinity than the South Australian populations.

A similar study on *Chloris virgata* Sw., a related species, reported that 9% of seeds germinated at 150 mM NaCl while no seeds germinated at 250 NaCl [16]. These results suggest that *C*. *truncata* may be more tolerant to saline conditions than *C*. *virgata*. This is pertinent because over 100,000 ha of land in Queensland is considered saline [17]. Thus, our results suggest that *C*. *truncata* has the ability to germinate in highly saline conditions, enabling it to colonize saline areas, and in addition to the general inhibitory effects of salinity on plant growth, the introduction of this saline-tolerant weed could further impact crop production.

### Water stress

**Fig 3:**
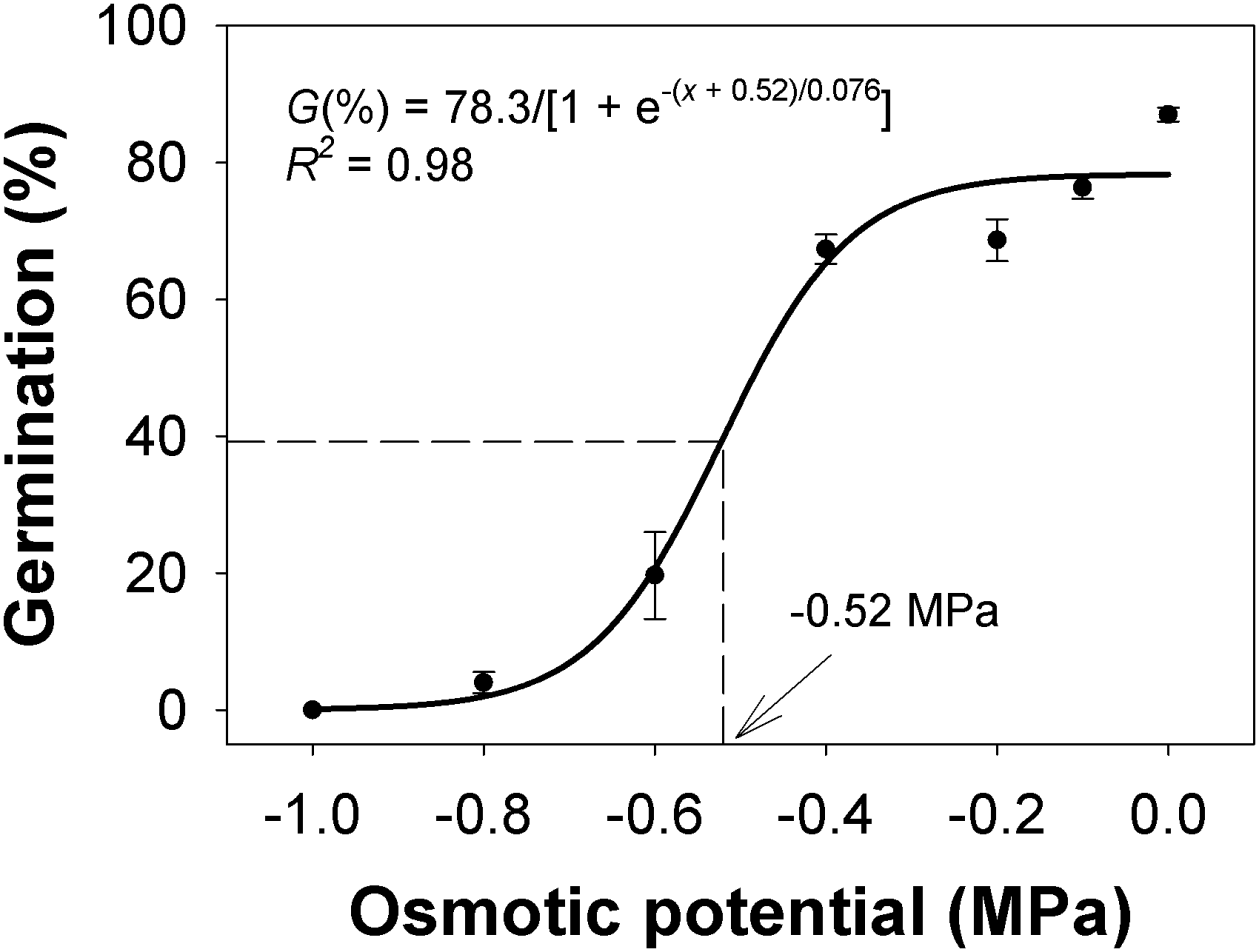
Effect of osmotic potential on seed germination of *Chloris truncata* after 21 d of incubation at 25/15°C day/night temperature. The line represents a three-parameter sigmoid model fitted to the data. The arrow shows the osmotic potential required to inhibit 50% germination. Vertical bars represent standard error of mean.

A sigmoid model was fitted to the germination response of *C*. *truncata* to osmotic potential (Fig 3). Our results showed that maximum germination (78%) was obtained under no water stress conditions, and that seed germination decreased from 78% to 65% as the osmotic potential of the environment decreased from 0 to -0.4 MPa. Whilst some seeds germinated at -0.8 MPa, germination was shown to be completely inhibited at -1.0 MPa. The fitted model suggested that an osmotic potential of -0.52 MPa will inhibit 50% of the maximum germination. This value was lower than reported for the South Australian populations. In the South Australian study, germination was inhibited by 50% at -0.27 MPa and 100% at -0.8 MPa [11]. These results suggest that Queensland populations of these seeds may be more tolerant to water stress conditions than the corresponding South Australian populations. Germination of *C*. *virgata* was completely inhibited at an osmotic potential of -0.6 MPa and the osmotic potential required for 50% reduction in germination was -0.09 MPa [16], indicating higher tolerance of *C*. *truncata* to water stress conditions.

The ability of *C*. *truncata* to germinate under moderate water stress conditions could enable it to have a competitive advantage due to earlier emergence than crop seedlings, and such conditions can occur in Queensland between rainfall events at the start of the summer season[18].

### pH

**Fig 4:**
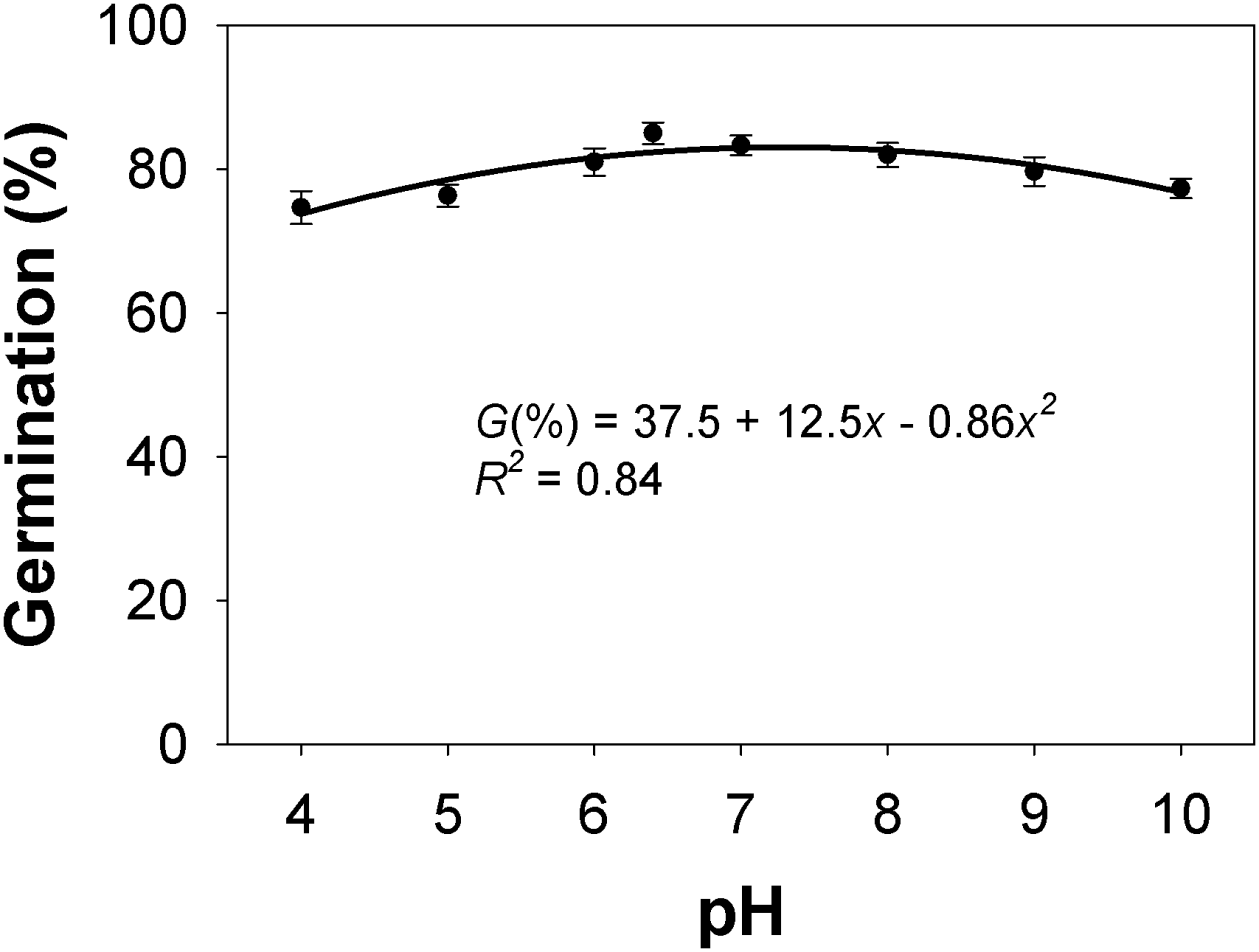
Effect of pH on seed germination of *Chloris truncata* after 21 d of incubation at 25/15°C day/night temperature. The line represents a quadratic model fitted to the data. Vertical bars represent standard error of mean.

There were no differences in seed germination between populations in response to changes in pH. A quadratic model was fitted to germination response to pH (Fig 4), and it is shown that seed germination ranged from 81 to 83% in the pH range 6 to 9. Although germination declined as the pH decreased from 6 (82% germination) to 4 (74% germination), and as the pH increased from 9 (81%) to 10 (77%), the high germination percentage over a broad range of normal pH conditions indicates that pH is not a limiting factor for *C*. *truncata* seed germination in most areas of Australia. Similar results were reported for *C*. *virgata* seeds collected from Queensland[16].

### Seed burial depth

**Fig 5:**
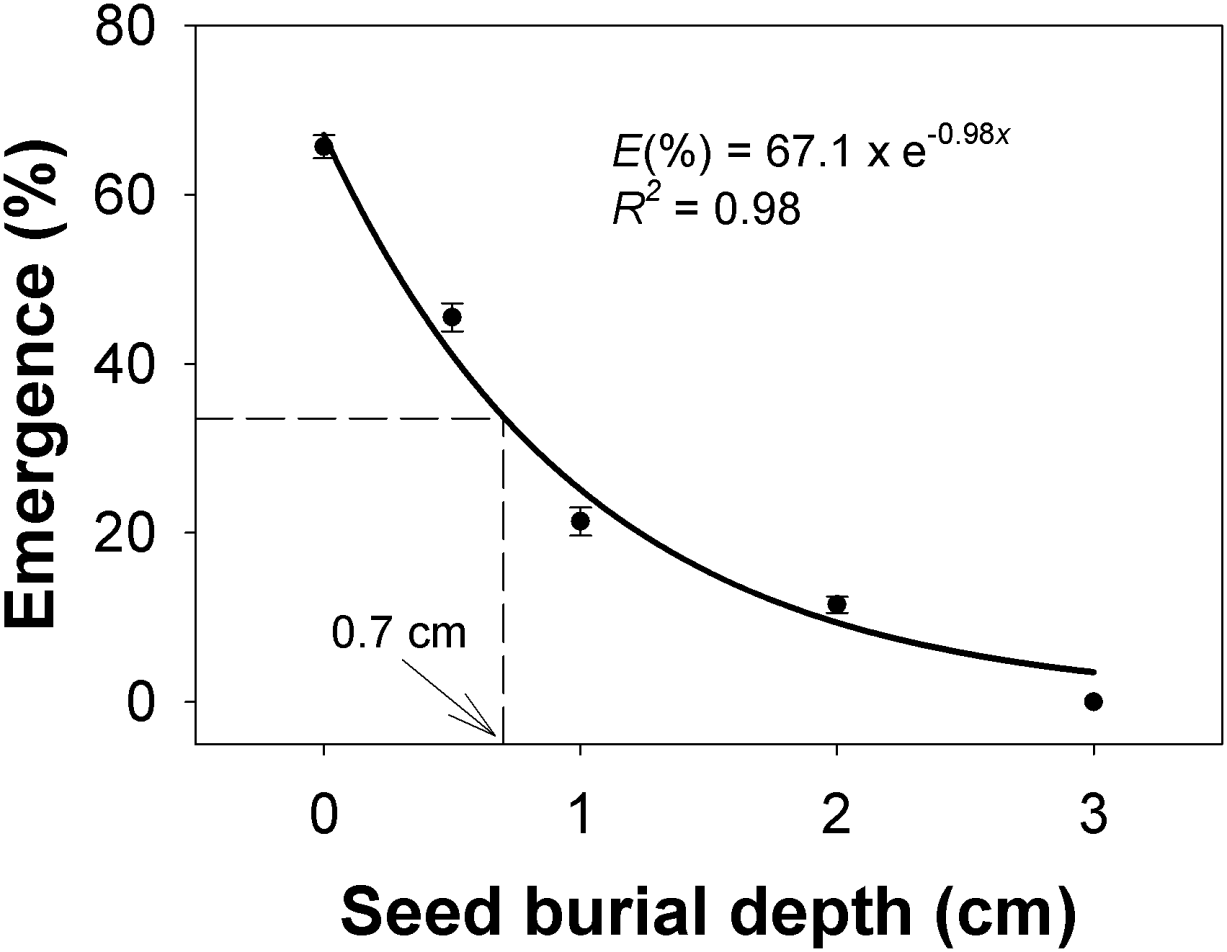
Effect of seed burial depth on seedling emergence of *Chloris truncata* after 21 d of incubation at 25/15°C day/night temperature. The line represents an exponential decay model fitted to the data. The arrow shows the seed burial depth required to inhibit 50% emergence. Vertical bars represent standard error of mean.

Seedling emergence under all burial depth conditions was not affected by the *C*. *truncata* populations, but seed burial depth greatly affected seedling emergence (Fig 5). Germination was 67% when seeds were placed on the soil surface, but as the burial depth increased, seedling emergence declined exponentially. Twenty-five percent of *C*. *truncata* seedlings emerged from 1 cm, and 9% seedlings emerged from 2 cm. There was no emergence from seeds buried deeper than 3 cm. Estimates from the fitted model indicated that the burial depth required for 50% inhibition of maximum emergence of *C*. *truncata* was 0.7 cm.

Seed germination on the soil surface was slightly lower than germination observed in Petri dishes in the light/dark condition. This difference could be due to poor soil-seed contact or limited availability of moisture for the seeds on the soil surface compared with on filter paper [19].

In the South Australian’ study, germination occurred only at the soil surface and no seedlings emerged from burial depths of 0.5 cm or deeper [11]. These results are in contrast with our study, in which seedlings emerged from up to 2 cm depth. Our results are consistent with the findings of the light experiment, in which more than 55% of seeds germinated in the dark. No seedlings emerged from the 3 cm burial depth, which could be due to death of the seeds or no germination. In this respect, large seeds such as *Echinichloa colona* (L.) Link are able to emerge from deep burial, but small seeds, such as *C*. *truncata*, may suffer from limited energy reserves which are needed to fuel hypocotyl elongation [20].

The phenomenon of greatest germination of seeds from near the soil surface suggests that farming practices that achieve shallow or no burial of weed seeds will promote greater emergence of *C*. *truncata*. This implies that widespread adoption of no-till and conservation agriculture systems in Australia is likely to favour *C*. *truncata* invasion [11]. A possible management option for farmers may therefore be a tillage operation that will bury the seeds below 3 cm (the maximum depth of emergence). A previous study also reported reduced emergence of *C*. *truncata* and *C*. *virgata* after tillage with harrow and offset discs as compared to no-till practice [21]. In this approach, subsequent tillage operations should be shallow to avoid the possibility of bringing the seed back to the soil surface; this is essential since up to 37% buried seeds were reported to be still viable after 14 months [11].

### Sorghum residue

**Fig 6:**
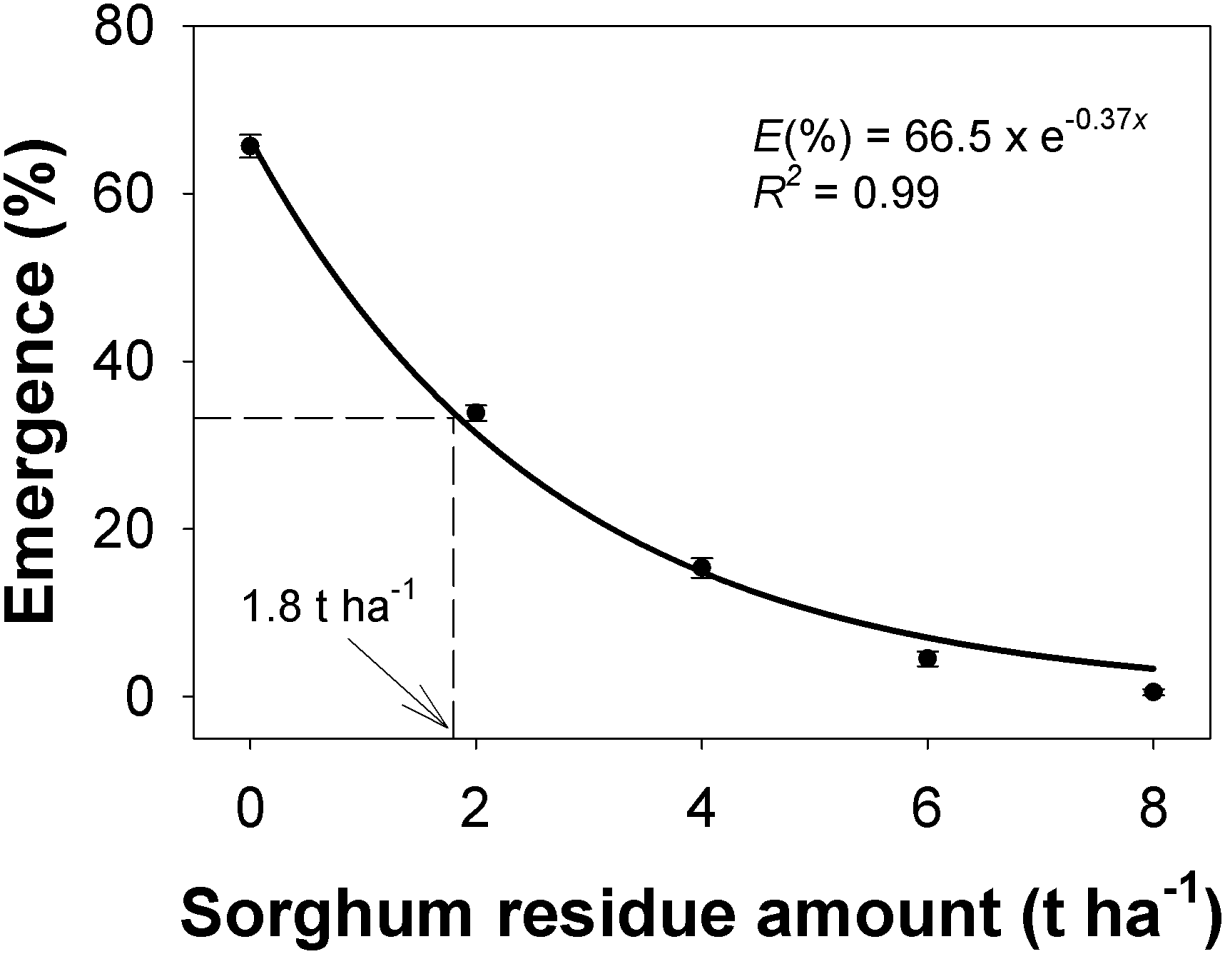
Effect of sorghum residue amount on seedling emergence of *Chloris truncata* after 21 d of incubation at 25/15°C day/night temperature. The line represents an exponential decay model fitted to the data. The arrow shows the sorghum residue amount to inhibit 50% emergence. Vertical bars represent standard error of mean.

Seedling emergence in response to sorghum residue amount was not affected by the source of *C*. *truncata* populations, but residue placed on the soil surface had a marked influence on *C*. *truncata* seedling emergence (Fig 6). Seedling emergence was greatest (67%) in the absence of residue, and as the amount of residue placed on the surface increased, emergence declined exponentially. There was only 7 to 15% emergence with residue of 4 to 6 t ha^-1^. The residue amount required to reduce emergence by 50% was estimated at 1.8 t ha^-1^.

Reduced seedling emergence due to addition of sorghum residue on the soil surface could be due to the effects of limited light penetration or the residue acting as a physical barrier [22, 23]. Reduced seedling emergence could also be attributed to the chemical effects of mulches on seed germination, as sorghum is known to have an allelopathic effect on seed germination [24].

In this controlled investigation, chopped sorghum residue was used which allowed uniform cover to be achieved. However, when unchopped material is used in normal field conditions, the residue needs to be anchored and also may not provide a uniform soil cover. In such situations, inhibition of seedling emergence may be less than reported in our study because of uncovered areas. Therefore, whilst we suggest that there is a need to integrate the use of crop residue retention with other management tools, it appears that crop residue retention in no-till farming systems could itself help farmers to reduce infestations of *C*. *truncata*.

## Conclusions

Seeds of *C*. *truncata* germinated at a wide range of temperature regimes, suggesting that the seeds can germinate throughout spring, winter and autumn seasons in Queensland, adding an extra level of necessary weed management post crop seedling emergence, which is not usually required in other more temperate areas.

A second observation was that while light clearly stimulated germination, it was not an absolute requirement. Seedlings were recorded as having emerged from a burial depth of up to 2 cm. The greatest emergence was observed for the seed placed on the soil surface, and this suggests that this weed will be favored in no-till farming systems. In this situation, most weed seeds will remain on or near the soil surface because the soil is not disturbed. The management of these surface seed banks would benefit from burying these seeds below their maximum depth of emergence (3 cm) and subsequently restricting further tillage to shallow operations to avoid returning seeds to germinable depths. In addition, retention of crop residues on the soil surface will increase shading of the soil, and by restricting available light to the seeds, will help in reducing infestation in conservation agriculture systems.

It is significant that purposeful variation of environmental factors did not affect germination of the two populations of *C*. *truncata* collected from different regions of Queensland. However, these populations from Queensland did responded differently to populations collected and studied in South Australia. The Queensland populations had higher germination in the dark, higher emergence from increasing burial depth, and were more tolerant to the effects of water and salt stress. This unexpected germination response with respect to seed origin strongly suggests that further studies need to be conducted to evaluate the effect of geographical environmental factors on developing seeds, with respect to germination and seedling emergence of *C*. *truncata* populations collected from throughout Australia.

## Materials and methods

### Seed collection

Mature seeds of two populations of *C*. *truncata* were collected in April 2017 in Queensland, Australia. These populations were collected from Gatton (S 27°30′20.5′′, E 152°18′33.5′′) and St. George (S 28°07′45.4′′, E 148°41′00.5′′) with the approximate distance between the two locations being 400 km. The authors confirm that the owner of the land gave permission to collect the weed seeds, as well as that the field studies did not involve endangered or protected species. Gatton and St. George receive an annual rainfall of 760 and 500 mm, respectively. Seeds were collected from approximately 50 plants per population. Seeds were cleaned, placed in plastic containers and stored in a laboratory until used in the experiment (July to October 2017).

### Germination tests

All experiments were conducted in the laboratory of the University of Queensland, Gatton, Australia. Seed germination of *C*. *truncata* was examined by placing 25 seeds of each population in a 9-cm diameter Petri dish containing two layers of filter paper. The filter paper was moistened with 5 ml of water or a treatment solution. Petri dishes were moistened with 5 ml of water or special treatment solution, then placed in sealed transparent plastic bags ready for the germination experiments. In all tests not involving temperature and light investigations, samples were incubated at standard fluctuating day/night temperatures of 25/15 °C in light/dark conditions. This 25/15 °C temperature regime was found to be the optimum condition for germination in the initial experiment on temperature and light variation. The photoperiod in the incubator was set at 12 h to coincide with the higher temperature interval. Seed germination was evaluated 21 days after the start of the experiment, and seeds were considered ‘germinated’ when the radical was approximately 2 mm long, and the amount of germination was reported as a percentage.

### Temperature and light

The effect of temperature and light on seed germination of *C*. *truncata* was determined by incubating seeds of both populations at four alternating day/ night temperatures (15/5, 20/10, 25/15, and 30/20 °C) in two light regimes [complete dark (24 h) and alternating light/dark (12/12 h)]. In the complete dark treatment, Petri dishes were wrapped in three layers of aluminum foil to ensure that no light penetrated to the seeds.

### Salt stress

The effect of salt stress on seed germination of *C*. *truncata* was determined by incubating seeds of both populations in sodium chloride (NaCl) solutions of 0 (control), 25, 50, 100, 150, 200, 250, and 300 mM.

### Water stress

The effect of water stress on seed germination of *C*. *truncata* was determined by incubating seeds of both populations at osmotic potentials of 0 (control), -0.1, -0.2, -0.4, -0.6, -0.8, and -1.0 MPa. These osmotic potential concentrations were prepared by dissolving polyethylene glycol 8000 in water as described in an earlier study [25].

### pH

The effect of pH on seed germination of *C*. *truncata* was determined by incubating seeds of both populations in buffer solutions of pH 4, 5, 6, 7, 8, 9, and 10. These solutions were prepared according to the method described in an earlier publication [26]. Unbuffered water (pH 6.4) was used as the control.

### Seed burial depth

The effect of seed burial depth on seedling emergence of *C*. *truncata* was determined by placing 50 seeds of each population on or in soil within 10 cm diameter pots. The seeds were placed on the soil surface (0 cm) or covered with the same soil to achieve soil burial depths of 0.5, 1, 2, and 3 cm. The soil used in the experiment had a pH of 7.2 and organic matter of 2.7%. The soil was sieved through a 3-mm sieve before conducting the experiment and it was confirmed that there was no background seed bank of *C*. *truncata* in the soil. Pots were placed in the incubator set at 25/15°C and subirrigated. The number of emerged seedlings was counted after 21 days and expressed as the percentage of total seeds used.

### Sorghum residue

The effect of sorghum residue amount on seedling emergence of *C*. *truncata* was determined by placing 50 seeds of each population on the soil surface within 10 cm diameter pots. These seeds were then covered with sorghum residue (leaves and stems of the variety MR-Buster) at rates equivalent to 0, 2, 4, 6, and 8 t ha^-1^. The amounts of sorghum straw used in this study reflect the quantity found in low and high yield systems in Australia. The soil used in this experiment was as described above for the seed burial depth experiment. Pots were placed in the incubator set at 25/15°C and subirrigated. Emerged seedlings were counted after 21 days and expressed as the percentage of total seeds used.

### Statistical analyses

All the experiments were conducted in a randomized complete block design with three replications and repeated over time. The second run was started within a month of the termination of the first run. There was no population by treatment interaction for any experiment and the population effect was also non-significant, so data were pooled across populations. ANOVA also indicated that there was no difference between the experiments conducted at different times and therefore, the data from the two experimental runs and two populations were combined for analysis. In the combined analysis, there were a total of 12 replications (2 populations × 2 runs × 3 replications).

The data of the temperature and light experiment were separated using the least significant difference (LSD) at p = 0.05 (GenStat, 16^th^ Edition). For other experiments, regression analysis was used (SigmaPlot 13.0). A three-parameter sigmoid model was used to analyze germination response to salt and water stress treatments:

$$G(\%)=Gmax/\{1+\text{exp}\;\left[-\frac{x-x50}{b}]\right\}$$ (1)

where, *G* (%) is the total germination at NaCl or osmotic potential *x*; *Gmax* is the maximum germination; *x50* is the NaCl or osmotic potential required for 50% inhibition of maximum germination; and *b* is the slope of the curve.

A quadratic model was fitted to the germination values obtained at different pH levels of the buffered solution:

$$G(\%)=a+bx+cx^{2}$$ (2)

where, a, b and c are constants.

An exponential decay curve of the form

E(%)=a×e(-bx) (3)

was fitted to seedling emergence values at different seed burial depths (cm) or residue amounts (t ha^-1^). In this equation, *E (%)* is emergence at burial depth or residue amount *x*, and *b* indicates the slope.
