# Correction: Germination ecology of *Chloris truncata* and its implication for weed management

**DOI:** 10.1371/journal.pone.0206870

**Published:** 2018-10-31

**Authors:** Bhagirath Singh Chauhan, Sudheesh Manalil, Singarayer Florentine, Prashant Jha

There are errors in the author affiliations. The affiliations should appear as shown here: Bhagirath Singh Chauhan^1^, Sudheesh Manalil^1, 2, 3^, Singarayer Florentine^4^, Prashant Jha^5^.

**1** The Centre for Crop Science, Queensland Alliance for Agriculture and Food Innovation (QAAFI), The University of Queensland, Gatton, Queensland, Australia, **2** School of Agriculture and Environment, Institute of Agriculture, The University of Western Australia, Perth, Australia, **3** Amrita University, Coimbatore, India, **4** Centre for Environmental Management, Faculty of Science and Technology, Federation University Australia, Mount Helen, Victoria, Australia, **5** Southern Agricultural Research Centre, Montana State University, Huntley, Montana, United States of America.
